# Language control is not a one-size-fits-all languages process: evidence from simultaneous interpretation students and the n-2 repetition cost

**DOI:** 10.3389/fpsyg.2015.01622

**Published:** 2015-10-21

**Authors:** Laura Babcock, Antonino Vallesi

**Affiliations:** ^1^Executive Function Laboratory, Department of Neuroscience, University of PadovaPadova, Italy; ^2^Cognitive Neuroscience Center, University of PadovaPadova, Italy

**Keywords:** n-2 repetition cost, simultaneous interpretation, inhibitory control, bilingual language control, language switching, multilingualism

## Abstract

Simultaneous interpretation is an impressive cognitive feat which necessitates the simultaneous use of two languages and therefore begs the question: how is language management accomplished during interpretation? One possibility is that both languages are maintained active and inhibitory control is reduced. To examine whether inhibitory control is reduced after experience with interpretation, students with varying experience were assessed on a three language switching paradigm. This paradigm provides an empirical measure of the inhibition applied to abandoned languages, the n-2 repetition cost. The groups showed different patterns of n-2 repetition costs across the three languages. These differences, however, were not connected to experience with interpretation. Instead, they may be due to other language characteristics. Specifically, the L2 n-2 repetition cost negatively correlated with self-rated oral L2 proficiency, suggesting that language proficiency may affect the use of inhibitory control. The differences seen in the L1 n-2 repetition cost, alternatively, may be due to the differing predominant interactional contexts of the groups. These results suggest that language control may be more complex than previously thought, with different mechanisms used for different languages. Further, these data represent the first use of the n-2 repetition cost as a measure to compare language control between groups.

## Introduction

Bilinguals face a large task daily. In every situation they encounter, they have to select the appropriate language to use. This task is complicated by the fact that both languages are always available, and therefore create interference (see Kroll et al., 2015 for a review). Yet, bilinguals rarely use the unintended language (Gollan et al., 2011) and mixing of languages is usually done with intention. Understanding how bilinguals accomplish this feat of language control has been the focus of a multitude of studies over the past three decades and several theoretical accounts have emerged. One theory posits a language specific selection mechanism in which only words from the target language are considered (Costa et al., 1999). Other accounts rely on activation and suggest that items in the target language receive additional activation from a language node (Poulisse and Bongaerts, 1994; La Heij, 2005; Colomé and Miozzo, 2010). Finally, some theories invoke inhibition as the primary mechanism used in language control (Dijkstra and van Heuven, 1998; Green, 1998). Among these accounts is the Inhibitory Control model which suggests that inhibition of the non-target language is reactive and proportional to the level of interference (Green, 1998). As this model has received considerable empirical support (e.g., Meuter and Allport, 1999; Price et al., 1999; Jackson et al., 2001; but see Costa and Santesteban, 2004; Costa et al., 2006 for contradictory evidence), an inhibition account has gained the most traction.

Though inhibition may underlie language control in most bilingual contexts (but see Costa and Santesteban, 2004 and Green and Abutalebi, 2013 for exceptional cases), inhibitory accounts face some difficulties when applied to perhaps the most demanding language control situation, namely, simultaneous interpretation (SI). SI requires an individual to comprehend a continuous stream of auditory material in one language and produce the same content in another language with a delay of a few seconds. This process entails a large degree of temporal overlap between the languages; one study estimated that about 70% of the time that interpreters are producing output in one language, they are also comprehending input in the other language (Chernov, 1994). This required simultaneity of languages limits the applicability of the widely accepted inhibitory account of bilingual language control to SI. If an interpreter were to inhibit the non-target source language, this might lead to a decrement in comprehension. Thus, language control during SI may rely on mechanisms other than inhibition.

Two previous studies of interpreters provide tentative support for a diminished reliance on inhibition during SI. The first study examined the speed of lexical access among professional translators (who had at least 2 years of interpretation experience) and non-translator bilinguals (Ibáñez et al., 2010). The task required participants to read and repeat sentences in Spanish and English. The authors included cognate and matched control words in the sentences to assess the simultaneous activation of the two languages. Faster processing of cognates than of control words would suggest active use of both languages. The translators, but not the bilinguals, showed faster processing of cognates than of control words in both languages. These results suggest that the translators maintained both languages active, while the bilinguals did not. Based on these results, the authors posited that translators do not rely on inhibition, but rather use other mechanisms that allow them to maintain two active languages.

This facility in actively maintaining two sets is further supported by a recent study of ours (Babcock and Vallesi, in press). Professional interpreters and matched multilinguals were assessed on a non-linguistic task-switching paradigm. The interpreters showed smaller mixing costs which gage the level of sustained control needed during the mixed-task blocks compared to the single-task blocks. Thus, the interpreters were better able to maintain the two task sets, likely due to experience maintaining two languages during SI.

These two studies provide a foundation of evidence that interpreters maintain two languages active during SI rather than relying solely on inhibition as a mechanism of language control. Neither study, however, directly assessed the use of inhibition. Therefore, in the present study, we sought to investigate the use of inhibition during language control in individuals with varying experience with SI. To this end, we examined students earning a Master in Conference Interpreting using a cross-sectional design with the aim of understanding how training in SI and recent practice with SI each affect the use of inhibitory control. The examination of both SI training and recent SI practice allowed us to consider the timescale of the potential effects. The participants were tested on a language switching paradigm as well as a task switching paradigm. The inclusion of the latter task allowed us to more directly assess the generality of advantages due to interpretation experience, such as those seen in our previous work (Babcock and Vallesi, in press). To quantify inhibition, we employed the n-2 repetition cost, a measure that assesses the cost of returning to a recently abandoned task (Mayr and Keele, 2000). This selection required three “tasks” to be used in each paradigm.

The n-2 repetition cost was first introduced by Mayr and Keele (2000) in an investigation of the inhibition applied to previous task sets during intentional shifts. The premise of the measure is that returning to a recently inhibited task should be more difficult and therefore cause a decrement in performance. To quantify this decrement the authors classified trials in a three task switching paradigm based on the task presented two trials previously (the *n-2* trial). On “n-2 repetition” trials participants performed the same task on trial *n* and trial *n-2* (e.g., Task A – Task B – Task A), while on “n-2 non-repetition” trials the tasks used on trials *n* and *n-2* differed (e.g., Task C – Task B – Task A). The finding that responses on n-2 repetition trials are slower and more error prone has been widely replicated (e.g., Arbuthnott and Frank, 2000; Schuch and Koch, 2003; Philipp and Koch, 2006; Arbuthnott, 2008; Houghton et al., 2009; Gade and Koch, 2012). More recently, several studies have extended the use of the n-2 repetition cost to language switching paradigms (Philipp et al., 2007; Philipp and Koch, 2009; Guo et al., 2013; Declerck et al., 2015). These studies have consistently found n-2 repetition costs, including in highly proficient bilinguals, confirming the use of inhibitory processes in language control.

The use of the n-2 repetition cost as the measure of interest has two benefits over the typical use of asymmetrical switch costs as the indicator of inhibitory control. First, it is possible that switch costs emanate from persistent activation of the previous task rather than persistent inhibition of the current task applied during the previous trial (Koch et al., 2010). Both of these accounts predict that switch costs are larger for the easier task than for the harder task. Thus, the presence of asymmetrical switch costs does not definitively signal the use of inhibition. In contrast, the n-2 repetition cost can be attributed singularly to the use of inhibition, as persistent activation would result in a benefit on n-2 repetition trials, which has not been evidenced. Thus, the n-2 repetition cost has been recognized as the empirical signature of inhibitory processes in task switching paradigms (Kiesel et al., 2010; Koch et al., 2010).

Second, the n-2 repetition cost quantifies the amount of inhibition applied to each task, whereas an asymmetry in switch costs indicates only the use (as opposed to the non-use) of inhibitory processes. The quantification of inhibition allows the analysis of factors that influence the level of inhibition applied to each specific task or language. The abovementioned studies that examined n-2 repetition costs in language switching were thus able to more directly examine the effect of language dominance on inhibition levels. Following the Inhibitory Control model, larger values would be expected for the more dominant language. However, no consistent pattern of n-2 repetition cost size was seen across the four studies, suggesting that factors beyond language dominance may contribute to inhibition levels.

We theorized that experience with SI would be one such factor. In particular that students with SI training and/or recent SI practice would exhibit smaller n-2 repetition costs in the language switching paradigm than students without training and experience. This result would suggest that language control during SI relies on mechanisms other than inhibitory control. Further, decreased n-2 repetition costs due to SI training alone would suggest that inhibitory control is modulated on a long-term scale, whereas decreased costs due to recent SI practice alone would suggest a shorter timescale of modulation. The effects of SI experience on n-2 repetition costs may also be expected to differ across the languages given the variety of results in previous studies and the asymmetry of language use during SI (the L1 is typically the target language and the L2 and L3 the source languages). Finally, if the reliance on other control mechanisms extends beyond language control, an extension which has been previously evidenced among bilinguals (e.g., Bialystok et al., 2008; Costa et al., 2008) and may be present in professional interpreters (Babcock and Vallesi, in press), smaller n-2 repetition costs among the experienced students would also be expected on the task switching paradigm.

## Materials and Methods

### Participants

Seventy students of Languages or Conference Interpreting at the University of Trieste participated in the study. The students were recruited to fall into one of four groups based on their training and recent practice with SI. The untrained-unpracticed group consisted of sixteen students (nine females) who had recently finished the coursework for a Triennale degree (equivalent to a Bachelor’s degree) in Languages. These students were not trained in, nor had they practiced, SI during their Triennale degree. Nineteen students (18 females) at the start of the Master in Conference Interpreting program formed the untrained-practiced group. At the time of testing these students had attended 1–2 months of courses in the program, thus they had recent practice with SI, but were not fully trained. Sixteen students (14 females) who had recently completed the coursework for the Master in Conference Interpreting program composed the trained-practiced group (one participant in this group had attended the Master’s program in Conference Interpreting at another university in Italy). These students were fully trained and actively practicing SI at the time of testing. Finally, the trained-unpracticed group consisted of nineteen students (13 females) who were working on their theses at the time of testing having completed the coursework for the Master in Conference Interpreting approximately 6 months earlier. These students were fully trained, but not actively practicing SI given their focus on thesis writing. All participants grew up speaking only Italian (or Italian dialects) and had no known neurological or psychiatric problems. They all reported normal color vision, which was confirmed with the Ishihara Color Vision Test (Ishihara, 1972). As biographical factors can influence measures of cognitive control, we confirmed (through a one-way ANOVA with four levels) that the four groups did not differ in intelligence (measured with Raven’s Advanced Progressive Matrices Series I; Raven et al., 1998) and socioeconomic status (measured with mother’s years of education, Gottfried et al., 2003; Noble et al., 2007; Stevens et al., 2009; Prior and Gollan, 2011; **Table 1**). Given the cross-sectional design of the study, however, it was not possible to match the four groups on age and years of education. All participants gave written informed consent in accordance with the Declaration of Helsinki and were compensated for their time. The study was approved by the Bioethical Committee of the Azienda Ospedaliera di Padova.

**Table 1:** Biographical and language characteristics of the four participant groups.

	Untrained, unpracticed (*N* = 16)	Untrained, practiced (*N* = 19)	Trained, practiced (*N* = 16)	Trained, unpracticed (*N* = 19)	*p*-value
Age (in years)	22.4 (1.7)	22.8 (1.8)	24.0 (1.3)	24.5 (1.0)	*p* < 0.001
Years of education	16.1 (0.7)	16.2 (0.4)	18.2 (0.5)	18.3 (0.7)	*p* < 0.001
Raven’s APM score	10.4 (1.7)	10.7 (1.6)	10.6 (1.8)	11.3 (0.9)	*p* = 0.334
Mother’s years of education	12.1 (3.3)	13.3 (2.6)	14.3 (4.2)	13.3 (3.6)	*p* = 0.423
Number of functional languages	3.6 (0.6)	3.4 (0.5)	3.6 (0.7)	3.6 (0.8)	*p* = 0.782
L2 reading	4.3 (0.4)	4.3 (0.5)	4.4 (0.5)	4.7 (0.5)	*p* = 0.014
L2 writing	3.8 (0.5)	3.9 (0.5)	4.1 (0.3)	4.3 (0.6)	*p* = 0.025
L2 speaking	3.9 (0.5)	4.1 (0.3)	4.2 (0.4)	4.4 (0.6)	*p* = 0.009
L2 understanding	4.2 (0.4)	4.2 (0.4)	4.3 (0.4)	4.7 (0.5)	*p* = 0.003
L3 reading	3.9 (0.3)	3.9 (0.6)	4.2 (0.4)	4.5 (0.6)	*p* = 0.003
L3 writing	3.4 (0.6)	3.4 (0.6)	3.5 (0.6)	3.7 (0.8)	*p* = 0.496
L3 speaking	3.2 (0.8)	3.4 (0.7)	3.4 (0.5)	3.6 (0.7)	*p* = 0.367
L3 understanding	4.1 (0.6)	3.8 (0.5)	4.0 (0.4)	4.3 (0.6)	*p* = 0.088

*Values reported are means with standard deviations in parentheses. *P*-values are from a one-way ANOVA with four levels. For one participant no language data was available and for another participant L2 self-ratings were missing.*

### Tasks and Procedure

Participants were tested individually in a sound-attenuated booth during a single session. During the session participants completed a language history questionnaire, Series I of Raven’s Advanced Progressive Matrices, the language switching paradigm, and the task switching paradigm (the order of the two switching paradigms was counterbalanced across participants).

#### Language History Questionnaire

Participants were asked to provide information about all of the languages they knew and/or studied. For each language they were asked to detail how and when they learned the language, including immersion experiences, as well as to provide a self-rating in the areas of reading, writing, speaking, and understanding on a 5-point Likert scale. Additionally, the participants completed a questionnaire developed to identify functional fluency for all their non-native languages. Functional fluency was operationalized as a B2 level or above in the Common European Framework of Reference for Languages (CEF). The questionnaire asked participants to give their CEF level and respond to eight yes-or-no questions which targeted the B1–B2 border (see Appendix in Supplementary Material for questionnaire items). The questionnaire contained two items for each of the four abilities (reading, writing, speaking, and oral comprehension), one item focused on academic usage and the other on personal usage. Participants were considered functionally fluent in languages for which they responded yes to seven or eight items. Finally, participants were also asked to evaluate how often they switched between languages within a conversation in the 2 years prior to testing using a 5-point Likert scale. This question was posed for the following seven situations: at home, with friends, at school/work, thinking, dreaming, talking to oneself, and expressing anger and affection. Additionally, participants in the trained-practiced group were asked to report how many hours they had practiced SI in the 2 months prior to testing.

The four groups did not differ in terms of their number of functionally fluent languages and self-ratings for L3 writing and speaking (see **Table 1**, for values). However, group differences were seen in the self-ratings across the L2 abilities and in L3 reading (*p*s ≤ 0.025; **Table 1**; L3 understanding showed a marginal difference). *Post hoc* Bonferroni-corrected tests revealed that the trained-unpracticed group gave higher self-ratings than the untrained-unpracticed group on all L2 abilities and L3 reading (*p*s ≤ 0.027). Additionally, the self-ratings for the trained-unpracticed group were higher than those of the untrained-practiced group on L2 reading and understanding, on L3 reading, and marginally on L3 understanding (*p*s ≤ 0.072). The trained-unpracticed group also reported higher self-ratings than the trained-practiced group on L2 understanding (*p* = 0.040). No other group differences in self-ratings were significant (*p*s ≥ 0.145). Finally, the groups differed in their frequency of code-switching only for thinking (*p* = 0.002), all other situations showed no difference (*p*s ≥ 0.115). *Post hoc* Bonferroni-corrected tests revealed that the untrained-practiced group code-switched more frequently when thinking than the trained-practiced group and the trained-unpracticed group (*p* = 0.002 and *p* = 0.029, respectively).

#### Raven’s Advanced Progressive Matrices

Non-verbal intelligence was measured using Series I of Raven’s Advanced Progressive Matrices (Raven et al., 1998). In this task, participants view patterns in a 3 × 3 matrix, each of which is missing a piece, and must choose the piece that completes the pattern from eight options. Series I contains 12 items and participants completed the task untimed, though most took 5–10 min.

#### Language Switching Paradigm

In this task, participants were asked to name digits in three languages. Italian, as the native language of all participants, was always included among the three languages. The remaining two languages were selected individually for each participant (languages used: Croatian, Dutch, English, French, German, Russian, and Spanish). These selections were based on the nature of their coursework for each language, their comfort level with each language, and the phonological similarity of the languages (with an attempt to avoid the following pairs of languages: Italian/Spanish and Dutch/German). Oﬄine the three languages were labeled as L1, L2, and L3. Italian was always L1; L2, and L3 were assigned based on the average self-rating across the four skill areas for each of the languages (in cases of parity, greater intensity of study and then greater comfort level were used).

**FIGURE 1:**
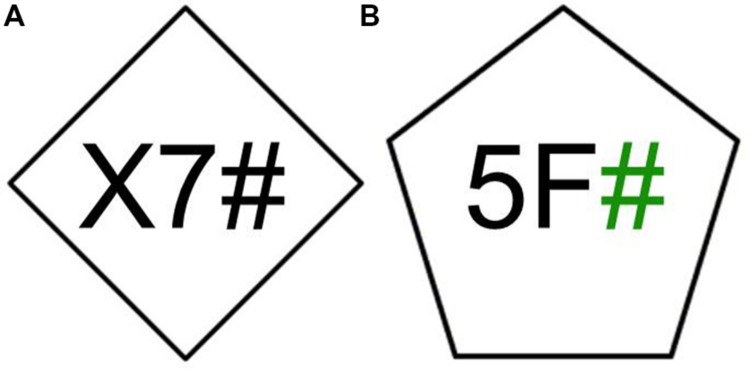
**Example items for **(A)** the language switching paradigm and **(B)** the task switching paradigm**.

In the task, participants viewed a series of stimuli composed of the letter X, the # sign, and a digit between 2 and 9 (the digit 1 was excluded due to high phonological similarity across the languages used). The X and # were not informative, but rather were included to match the visual complexity of the stimuli used in the task switching paradigm. The stimuli components were black and were presented with equal probability in each of the six possible orders (e.g., X#2, 2#X). Participants were asked to name aloud the digit in their L1, L2, or L3 according to the cue presented. Cues were black frames surrounding the stimulus in the shape of a diamond, a hexagon, and a triangle (see **Figure 1** for an example item). Graphic cues were chosen because they have been previously associated with larger n-2 repetition costs (Houghton et al., 2009; Guo et al., 2013). The cue-language pairings were counterbalanced across participants. A visual reminder of these pairings was placed below the computer screen to decrease working memory requirements and ensure correct assignment throughout the task. Each stimulus was categorized as either an n-2 repetition or non-repetition trial; the difference between these trial types quantifies inhibition. On n-2 repetition trials, the language used on the current trial was the same as that used on the n-2 trial (e.g., English – Italian – English). Thus participants were returning to a recently inhibited language on these trials. Conversely, on n-2 non-repetition trials, the current language differed from that used on the n-2 trial (e.g., French – Italian – English). Immediate language repetitions were excluded from the task design since their presence has been associated with a decrease in n-2 repetition cost (Philipp and Koch, 2006). Consequently, n-2 non-repetition trials made use of all three languages and n-2 repetition trials of two languages.

Each trial began with a 500 ms blank screen followed by cue presentation. The target stimulus appeared inside the cue 100 ms later. A short cue-to-stimulus interval was employed to enhance n-2 repetition costs (Philipp et al., 2007; Guo et al., 2013). The stimulus and cue remained onscreen until 200 ms after the participants started their vocal response, at which point the trial ended. The onset of vocal responses was recorded with a voice key. Before the task instructions were given, a microphone sensitivity test was completed in which the participant pronounced the numbers 2–9 in each of their three languages. The microphone was adjusted and the test repeated until all possible responses successfully triggered the voice key. The experiment began with a practice session consisting of 30 trials, which could be repeated until the participant felt comfortable with the task paradigm (10 participants repeated the practice once). The experimental session consisted of six blocks, each with 120 trials. After every block there was a break and participants could initiate the next block with a button press. The sequence of trials was pseudo-randomized for each participant according to the following restrictions. There were an equal number of n-2 repetition and non-repetition trials, as well as an equal number of trials for each language, divided approximately evenly between n-2 repetition and non-repetition trials. Further, in each block of 120 trials, each digit was presented 15 times, five times in each language. Immediate language repetitions and immediate digit repetitions were excluded from the task design. Finally, a digit could not appear with the current language if it had been used on the most recent trial using that language.

Participant responses were recorded using a digital recorder. The recording was used oﬄine to code the accuracy of responses. To aid in the alignment of responses and trial number during oﬄine coding, a 100 ms beep during cue presentation, unheard by participants, was fed directly to the digital recorder. Responses were coded for the accuracy of the response (i.e., the correct number produced in the correct language) and for false starts, when a sound other than a response triggered the voice key (e.g., a cough, a speech filler, a corrected response from the previous trial).

#### Task Switching Paradigm

The task switching paradigm was created to be as similar as possible to the language switching paradigm. This choice allowed any potential differences in results between the two paradigms to be attributed to specific between-language switching mechanisms, rather than differences in task design. The stimuli in this paradigm were composed of a black digit (2–9), a black letter (A–H), and a colored # sign (black-*nero*, gray-*grigio*, blue-*blu*, green-*verde*, red-*rosso*, yellow-*giallo*, pink-*rosa*, purple-*viola*; color of # sign-*expected response in Italian*). To allow for comparability in the stimulus set size between this paradigm and the language switching paradigm, eight composite stimuli were created for each participant. The composite stimuli grouped a number, a letter, and a color which appeared together throughout the experiment with equal probability in each of the six possible orders (e.g., letter-number-#, number-#-letter). Participants were asked to name aloud in Italian the digit, the letter, or the color of the # sign according to the cue presented. Cues were black frames surrounding the stimulus in the shape of a square, a circle, and a pentagon (see **Figure 1** for example item). The cue-task pairings were counterbalanced across participants. A visual reminder of these pairings was visible throughout the experiment. As in the language switching task, each stimulus was classified as either an n-2 repetition trial (e.g., number – color – number) or an n-2 non-repetition trial (e.g., letter – color – number). The details of the task presentation, pseudo-randomization, and oﬄine coding were identical to those used in the language switching paradigm. During the microphone sensitivity test, participants named the eight digits, letters, and colors presented in the task. This test was additionally used to check that the participants correctly named the colors. As with the language switching paradigm, participants could repeat the practice session until they felt confident in the task (six participants repeated the practice once).

## Results

### Language Switching Paradigm

Data from one participant (from the trained-unpracticed group) were excluded due to a disruption during the task. Non-parametric tests were used to analyze the accuracy data given that these data were not normally distributed. The Kruskal–Wallis test was used to compare accuracy between the four groups and the Wilcoxon signed-ranked test was used to compare the conditions. Response time (RT) data were analyzed using a mixed effects four-way ANOVA with trial type (n-2 repetition, n-2 non-repetition) and language of the current trial (L1, L2, L3) as within-subjects factors and training (untrained, trained) and recent SI practice (unpracticed, practiced) as between-subjects factors. For these analyses error trials and the two trials following an error were excluded, to ensure the correct trial type assignment. Trials with a false start were also excluded since RT was not an accurate reflection of performance in these trials. Additionally, for each participant, trials with an RT more than three standard deviations from their individual mean were excluded. Finally, responses that were faster than 200 ms and slower than 3500 ms were excluded. These data trimming procedures resulted in the exclusion of 12.1% of all trials. Reported results reflect a Greenhouse–Geisser correction. Performance data for the language switching paradigm are reported in **Table 2**.

**Table 2:** Response times (RT) and accuracy rates (ACC) on the language switching paradigm by group.

		Untrained, unpracticed		Untrained, practiced		Trained, practiced		Trained, unpracticed	
		RT (ms)	ACC (%)	RT (ms)	ACC (%)	RT (ms)	ACC (%)	RT (ms)	ACC (%)
L1	n-2 repetition	859 (139)	97.8 (2.5)	894 (200)	97.6 (1.6)	933 (182)	97.5 (2.5)	905 (247)	97.3 (2.2)
	n-2 non-repetition	838 (141)	97.9 (2.0)	884 (183)	97.5 (2.3)	923 (177)	97.7 (2.2)	858 (224)	97.9 (2.1)
	n-2 repetition cost	20 (53)	0.1 (2.7)	11 (54)	-0.2 (1.6)	10 (50)	0.2 (2.0)	47 (44)	0.7 (2.4)
L2	n-2 repetition	967 (200)	96.7 (2.9)	1000 (225)	95.8 (3.3)	1060 (177)	97.8 (1.9)	971 (250)	95.3 (2.5)
	n-2 non-repetition	901 (175)	96.9 (3.0)	948 (201)	97.1 (1.9)	1014 (171)	97.4 (1.9)	949 (232)	96.1 (3.5)
	n-2 repetition cost	66 (57)	1.2 (2.8)	51 (48)	1.2 (2.8)	46 (43)	-0.4 (2.0)	22 (55)	0.8 (3.3)
L3	n-2 repetition	959 (175)	96.9 (2.8)	995 (175)	95.9 (3.2)	1064 (205)	97.2 (2.3)	998 (238)	95.7 (3.2)
	n-2 non-repetition	918 (176)	97.3 (2.5)	941 (153)	96.7 (2.9)	1017 (182)	96.9 (2.5)	940 (209)	96.7 (2.9)
	n-2 repetition cost	42 (37)	0.5 (2.3)	54 (35)	0.9 (2.8)	47 (44)	-0.3 (1.5)	58 (56)	0.4 (2.7)

*Values reported are means with standard deviations in parentheses.*

Analyses on accuracy revealed no overall difference between the groups (*p* = 0.197). Further, there were no differences between the groups when considering each language and trial type separately (*p*s ≥ 0.089). Across the groups, accuracy was higher on n-2 non-repetition trials than on n-2 repetition trials (Wilcoxon *T* = 1227, *Z* = 2.025, *p* = 0.043). Additionally, accuracy was higher when responding in L1 than in L2 and L3 (Wilcoxon *T* = 566, *Z* = 3.836, *p* < 0.001 and Wilcoxon *T* = 654, *Z* = 3.309, *p* = 0.001, respectively), with no difference between L2 and L3 (*p* = 0.650).

**FIGURE 2:**
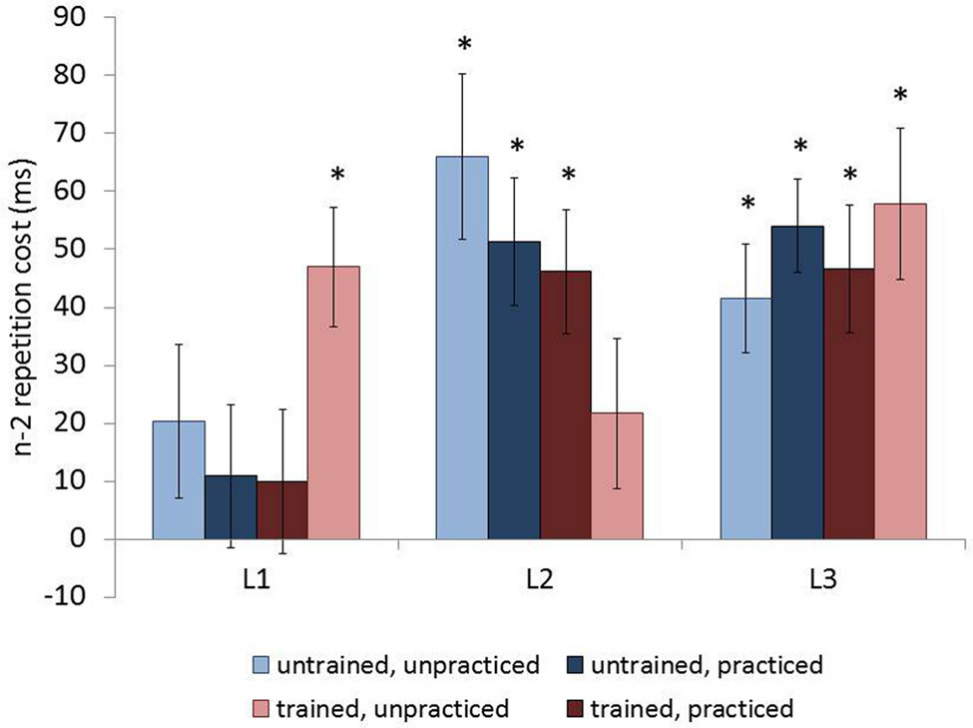
**N-2 repetition costs for each language by group on the language switching paradigm.** Blue bars represent groups without training, red bars represent groups with training; light bars represent groups without recent practice, dark bars represent groups with recent practice. Error bars represent the standard errors of the mean. ^∗^*p* < 0.05 for the comparison of n-2 repetition and non-repetition trials.

The RT analysis showed a main effect of trial type [*F*(1,65) = 91.794, *p* < 0.001, $\eta_{p}^{2}$ = 0.585]. Responses were faster to n-2 non-repetition trials than n-2 repetition trials, demonstrating the expected n-2 repetition cost (see **Table 2** for values). The main effect of language was also significant [*F*(1.831,119.044) = 33.125, *p* < 0.001, $\eta_{p}^{2}$ = 0.338]. *Post hoc t*-tests (evaluated at α = 0.017 to correct for multiple comparisons) revealed that responses to L1 were faster than to L2 and L3 [*t*(68) = 6.179, *p* < 0.001 and *t*(68) = 7.879, *p* < 0.001, respectively], with no difference between L2 and L3 (*p* = 0.813). Trial type and language also showed a significant interaction [*F*(1.964,127.675) = 8.878, *p* < 0.001, $\eta_{p}^{2}$ = 0.120]. Through *post hoc t*-tests (evaluated at α = 0.017 to correct for multiple comparisons), the n-2 repetition cost in L1 was shown to be smaller than in L2 and L3 [*t*(68) = 2.951, *p* = 0.004 and *t*(68) = 3.738, *p* < 0.001, respectively], which did not differ (*p* = 0.515). There were no main effects due to training (*p* = 0.327) or recent practice (*p* = 0.260), nor was there a significant interaction between these two factors (*p* = 0.753). However, these group level factors did show significant interactions with the trial type and language interaction. The three-way interaction of trial type, language, and training was significant [*F*(1.964,127.675) = 3.735, *p* = 0.027, $\eta_{p}^{2}$ = 0.054], as was the four-way interaction of trial type, language, training, and recent SI practice [*F*(1.964,127.675) = 3.361, *p* = 0.039, $\eta_{p}^{2}$ = 0.049, **Figure 2**]. No other interactions were significant (*p*s ≥ 0.115).

To understand the provenance of the four-way interaction we evaluated which language-group pairings showed significant n-2 repetition costs using twelve paired *t*-tests (evaluated at α = 0.004 to correct for multiple comparisons). In L1, the n-2 repetition cost was not significant in the untrained-unpracticed, untrained-practiced, and trained-practiced groups (*p* = 0.149, *p* = 0.392, and *p* = 0.435, respectively). The trained-unpracticed group, however, did show a significant repetition cost (*p* < 0.001). In L2, the opposite pattern was seen, that is, significant n-2 repetition costs for the untrained-unpracticed, untrained-practiced, and trained-practiced groups (*p*s ≤ 0.001), but no significant repetition cost in the trained-unpracticed group (*p* = 0.112). In L3, all four groups showed a significant n-2 repetition cost (*p*s ≤ 0.001).

To more directly assess the influence of recent practice with SI on the size of the n-2 repetition cost, we tested for correlations between the n-2 repetition costs in L1, L2, and L3 and the amount of practice values supplied by trained-practiced group. Marginal positive correlations between SI practice and n-2 repetition cost were seen in L2 and L3 (*r* = 0.464, *p* = 0.070 and *r* = 0.453, *p* = 0.078, respectively), but not in L1 (*p* = 0.362).

As the factors related to experience with SI did not unequivocally explain the variation of n-2 repetition costs, we additionally explored the role of other language characteristics. The self-ratings in L2 and L3 of all participants (L1 self-ratings were not considered due to lack of variability) were tested for correlations with the n-2 repetition costs in L1, L2, and L3. The n-2 repetition cost in the L2 was negatively correlated with self-rated speaking and understanding in L2 (*r* = –0.237, *p* = 0.053 and *r* = –0.316, *p* = 0.009, respectively), while the n-2 repetition cost in L3 showed a marginal positive correlation with self-rated reading in L3 (*r* = 0.216, *p* = 0.076). All other correlations were non-significant (*p*s ≥ 0.107). In addition to self-ratings, the reported frequencies of switching in seven situations over the 2 years prior to testing (see Language History Questionnaire section for details) were tested for correlations with the n-2 repetition costs. No correlations were significant (*p*s ≥ 0.148). The above-reported correlation analyses were not corrected for multiple comparisons as they were of an exploratory nature. Given this fact, these findings should be viewed with some caution.

### Task Switching Paradigm

Data from three participants were excluded from all analyses of this task. Two participants (one from each of the untrained-practiced and trained-unpracticed groups) were identified as extreme outliers within their groups based on their accuracy rate (more than 3 interquartile ranges below the 1st quartile). An additional participant (from the untrained-practiced group) was excluded due to difficulties coding her data oﬄine. The exclusion of these participants did not change the results of the comparisons between the groups on the biographical and language characteristics mentioned above.

Non-parametric tests were used to analyze the accuracy data given that these data were not normally distributed. The Kruskal–Wallis test was used to compare between the four groups and the Wilcoxon signed-ranked test was used to compare the conditions. The data trimming procedures (10.4% of all trials removed) and analyses performed on the RT data were identical to those used for the language switching task. Performance data for the task switching paradigm are reported in **Table 3**.

**Table 3:** Response times (RT) and accuracy rates (ACC) on the task switching paradigm by group.

		Untrained, unpracticed		Untrained, practiced		Trained, practiced		Trained, unpracticed	
		RT (ms)	ACC (%)	RT (ms)	ACC (%)	RT (ms)	ACC (%)	RT (ms)	ACC (%)
Number	n-2 repetition	1115 (217)	97.1 (2.6)	1136 (260)	97.6 (2.5)	1202 (243)	97.4 (2.6)	1095 (225)	96.9 (2.7)
	n-2 non-repetition	1037 (193)	97.6 (1.8)	1017 (228)	98.2 (1.6)	1101 (224)	98.9 (0.9)	1007 (221)	98.1 (1.6)
	n-2 repetition cost	78 (76)	0.4 (2.0)	119 (65)	0.7 (1.9)	101 (52)	1.6 (2.3)	88 (47)	1.2 (2.2)
Letter	n-2 repetition	1128 (237)	96.9 (2.2)	1132 (257)	97.6 (2.0)	1154 (243)	98.4 (1.4)	1126 (214)	96.7 (2.2)
	n-2 non-repetition	1043 (205)	96.4 (2.5)	1057 (259)	97.9 (1.4)	1082 (268)	98.6 (2.0)	1023 (191)	98.0 (1.4)
	n-2 repetition cost	85 (70)	-0.5 (1.8)	75 (46)	0.4 (1.5)	72 (58)	0.2 (1.7)	103 (78)	1.3 (1.6)
Color	n-2 repetition	1133 (219)	97.4 (1.7)	1148 (259)	97.8 (1.4)	1188 (187)	97.5 (1.9)	1174 (192)	96.3 (3.2)
	n-2 non-repetition	1022 (219)	97.7 (2.5)	1049 (255)	98.6 (1.6)	1094 (165)	98.0 (2.1)	1073 (192)	95.8 (3.5)
	n-2 repetition cost	110 (47)	0.2 (1.8)	98 (52)	0.8 (1.8)	94 (62)	0.4 (2.2)	101 (86)	-0.5 (3.2)

*Values reported are means with standard deviations in parentheses.*

Analyses on accuracy revealed no overall difference between the groups (*p* = 0.389). Further, there were no differences between the groups when considering each task and trial type separately (*p*s ≥ 0.112). Across the groups, accuracy was higher on n-2 non-repetition trials compared to n-2 repetition trials (Wilcoxon *T* = 1407, *Z* = 3.029, *p* = 0.002). There were no differences in accuracy between the three tasks (*p*s ≥ 0.612). The RT analysis showed a main effect of trial type [*F*(1,63) = 419.777, *p* < 0.001, $\eta_{p}^{2}$ = 0.870] with faster responses to n-2 non-repetition trials than n-2 repetition trials (see **Table 3** for values). Additionally, the interaction of trial type, task, and recent practice showed a marginal effect [*F*(1.996,125.753) = 2.61, *p* = 0.078, $\eta_{p}^{2}$ = 0.040]. However, *post hoc t*-tests (evaluated at α = 0.017 to correct for multiple comparisons) comparing the n-2 repetition cost on each task between the practiced and unpracticed groups showed no effects (*p*s ≥ 0.070). No other effects or interactions were significant (*p*s ≥ 0.269).

### Correlations between the Paradigms

Correlational analyses of the RTs and n-2 repetition costs from the two paradigms were computed to further examine the generalizability of modulations of language control mechanisms. All four participants who were excluded in either of the paradigms were likewise excluded from these analyses. Correlational analyses were performed between the overall RT on the task switching paradigm (as no differences were seen between the tasks) and the average RT for each language on the language switching paradigm. All three tests yielded a large correlation (*r*s ≥ 0.618, *p*s < 0.001). Analogous correlational analyses performed using the n-2 repetition cost yielded no significant correlations (*r*s ≤ 0.141, *p*s ≥ 0.260).

## Discussion

The aim of the present study was to begin to understand how languages are controlled during SI by investigating the hypothesis that inhibitory control does not play a large role. Multilingual students with or without SI training and recent SI practice completed language switching and task switching paradigms that included three languages or tasks, thus allowing the computation of n-2 repetition costs, which provide a purer measure of inhibition during switching paradigms than switch costs.

The results did not show a general reduction in inhibition across the languages due to SI training, recent SI practice, or a combination of these two factors. Instead a more complex pattern of inhibition levels was evidenced across the groups and languages. In L1, all groups except the trained-unpracticed group exhibited negligible n-2 repetition costs. The reverse pattern was revealed in L2 with the trained-unpracticed group showing a negligible repetition cost and the other three groups a reliable cost. Finally, in L3, all four groups showed a reliable cost. Though group differences were seen in the L1 and L2, they cannot be explained by factors related to SI experience, given that the most experienced (trained-practiced) and the least experienced (untrained-unpracticed) groups patterned together. Thus, the present data do not furnish support for the use of mechanisms other than inhibitory control during SI.

However, they also do not provide strong support against this hypothesis. A change in language control processes may require greater cumulative experience with SI than what students of interpretation have. Further, students of interpretation may not spend enough time on SI daily to maintain this change. For these reasons, an examination of professional interpreters would be needed to challenge the hypothesis. Additionally, it may be prudent to investigate language control not only in productive language, but also in receptive language. SI requires the comprehension of two languages, but the production of only one. Thus, if language control is separable into receptive and productive modules, as has been posited by some models to accommodate SI (Grosjean, 1997; Christoffels and de Groot, 2005), an absence of inhibitory control would be more strongly predicted in the control of receptive language. Therefore, future studies may wish to examine professional interpreters on both productive and receptive language control.

Though the present data did not clarify the use of inhibitory control in SI, they do speak more generally to the complex nature of language control. The similar pattern of n-2 repetition costs seen across the untrained-unpracticed, untrained-practiced, and trained-practiced groups may be explained by language experience factors that unite these groups and distinguish them from the trained-unpracticed group. One such factor is L2 proficiency on which the trained-unpracticed group reported the highest self-ratings. This factor may, indeed, explain the group differences in n-2 repetition cost seen in the L2, though not in the L1, given the association between oral L2 proficiency and inhibition in the L2. Interestingly, this association suggests that inhibition of the L2 decreases with increasing proficiency, while a reactive inhibition account would predict the opposite pattern. It should, however, be noted that the correlational analyses were exploratory and therefore the influence of L2 proficiency on inhibition needs to be investigated further.

In addition to L2 proficiency, the trained-unpracticed group may also differ from the other groups in their prevailing language context. The untrained-unpracticed, untrained-practiced, and trained-practiced groups were all actively engaged in courses and school-life at the time of testing. The trained-unpracticed students, on the other hand, were engaged in the solitary task of thesis writing. Notably, the reported frequency of code-switching across the groups was highest for the “at school” situation. Thus, if we consider the language contexts presented by Green and Abutalebi (2013), the untrained-unpracticed, untrained-practiced, and trained-practiced groups may have been predominantly in a code-switching context, while the trained-unpracticed students were more often in single- or dual-language contexts. The negligible inhibition in L1 among the untrained-unpracticed, untrained-practiced, and trained-practiced groups may then be explained by the use of language control mechanisms other than inhibition which define code-switching contexts. The trained-unpracticed students, on the other hand, were in language contexts that rely on inhibition for language control and therefore showed reliable inhibition in L1. Thus, the level of inhibition applied to the L1 seems to be best explained by the different interactional contexts. This explanation, however, does not generalize to the pattern seen in the L2.

These data may then suggest that each language can be controlled by a different mechanism and that the mechanisms recruited depend on the interaction of multiple factors. This understanding of language control may explain the variety of patterns seen in the previous studies of language switching which employed the n-2 repetition cost. As those studies did not compare between groups or provide detailed language experience information, they cannot further confirm the influence of proficiency and interactional context on the use of inhibitory control. Future studies should further explore this novel suggestion making full use of n-2 repetition costs and detailed language experience and usage information.

This modulation of control mechanisms based on language experience factors, however, does not appear to extend beyond control between the languages. No influence of group membership was seen on the size of the n-2 repetition costs in the task switching paradigm. On one view this may be unsurprising given that the changes in inhibitory control on the language paradigm appeared to target specific languages rather than apply across the languages. However, the task switching paradigm was conducted entirely in the L1 and thus a pattern of n-2 repetition costs similar to that seen in the L1 would be expected if the modulations in inhibitory control were generalizable. The absence of such a pattern in the task switching paradigm supports a dissociation between the control processes within- and between-languages. This dissociation is additionally supported by the lack of a correlation between the n-2 repetition costs across the two paradigms; a result that has also been evidenced by Branzi (unpublished). Thus, it seems that changes in the usage of inhibitory processes during language control may be extremely localized and not generalizable, a conclusion which is in line with previous work (e.g., Calabria et al., 2012).

The present study examined inhibitory control usage by examining the n-2 repetition costs during language and task switching paradigms. In a novel expansion of the use of this measure, we employed it to make comparisons between groups. Though the present data did not contribute to our understanding of language control during SI in a simple way, they did suggest that language control is a complex process which may rely on different mechanisms for different languages. Further, the present data lend support to the supposition that between-language control is, at least partially, separate from more general control.

## Author Contributions

LB designed the research project. LB and AV designed the tasks. LB collected and analyzed the data. LB and AV wrote the manuscript.

## Conflict of Interest Statement

The authors declare that the research was conducted in the absence of any commercial or financial relationships that could be construed as a potential conflict of interest.
