# Supplementary material for: Language control is not a one-size-fits-all languages process: evidence from simultaneous interpretation students and the n-2 repetition cost
Source: Front Psychol. 2015 Oct 21;6:1622. doi: 10.3389/fpsyg.2015.01622 (PMC4612644; doi:10.3389/fpsyg.2015.01622)
Supplement: Supplementary file 1 [file Data_Sheet_1.DOCX]

**Appendix:** Functionally Fluent Questionnaire

*English version*

Please list all the foreign languages you have studied or know. For each language please answer the following questions with that language in mind.

1. Do you know the A1, A2, B1 etc. system of classifying language levels? If yes, what is your level in this language?
2. Could you discuss a topic in which you are not an expert, such as politics, in this language?
3. Can you understand news programs in this language?
4. Could you read a novel or short story for pleasure in this language?
5. Could you tell a story about events in the past, present, and future to a group of people in this language?
6. Could you write a letter in this language to a friend about an important event in your life and how it affected you?
7. Would you be able to understand an announcement about a cancelled train in this language and follow the directions given about where to refund or change your ticket?
8. Could you write an essay in this language on a work of literature?
9. Could you understand a textbook passage on your field of study in this language?

*Italian version*

Per favore elenca le lingue straniere che hai studiato o che conosci. Per ogni lingua, per favore rispondi alle seguenti domande riferendoti a quella lingua.

1. Conosci il sistema di classificazione A1, A2, B2, ecc. che indica il livello di conoscenza di una lingua? Se si, quale e’ il tuo livello in questa lingua?
2. Potresti parlare di un tema del quale non sei esperto, come la politica, in questa lingua?
3. Potresti comprendere un telegiornale in questa lingua?
4. Potresti leggere un romanzo o un breve racconto per passatempo in questa lingua?
5. Potresti raccontare una storia su un evento del passato, uno del presente e uno del futuro a un gruppo di persone in questa lingua?
6. Potresti scrivere una lettera in questa lingua ad un amico riguardo un evento importante della tua vita e di come ti ha influenzato?
7. Saresti capace di comprendere un annuncio riguardo un treno cancellato in questa lingua e seguire le istruzioni date su dove farti rimborsare o cambiare il biglietto?
8. Potresti scrivere un saggio in questa lingua su un brano di letteratura?
9. Potresti comprendere un passaggio di un libro di testo nel tuo campo di interesse in questa lingua?
